# A facile approach to a silver conductive ink with high performance for macroelectronics

**DOI:** 10.1186/1556-276X-8-296

**Published:** 2013-06-25

**Authors:** Yu Tao, Yuxiao Tao, Biaobing Wang, Liuyang Wang, Yanlong Tai

**Affiliations:** 1 School of Materials Science and Engineering, Changzhou University, Changzhou 201326, People’s Republic of China; 2 Department of Biomedical Engineering, University of California Davis, Davis, CA 95616, USA

**Keywords:** Organic silver conductive ink (OSC ink), Conductive pattern, Formula mechanism, Antenna pattern

## Abstract

An unusual kind of transparent and high-efficiency organic silver conductive ink (OSC ink) was synthesized with silver acetate as silver carrier, ethanolamine as additive, and different kinds of aldehyde-based materials as reduction agents and was characterized by using a thermogravimetric analyzer, X-ray diffraction, a scanning electron microscope, and a four-point probe. The results show that different reduction agents all have an important influence on the conductive properties of the ink through a series of complex chemical reactions, and especially when formic acid or dimethylformamide was used as the reduction agent and sintered at 120°C for 30 s, the resistivity can be lowered to 6 to 9 μΩ·cm. Furthermore, formula mechanism, conductive properties, temperature, and dynamic fatigue properties were investigated systematically, and the feasibility of the OSC ink was also verified through the preparation of an antenna pattern.

## Background

Most research efforts in macroelectronics have opened the door for the manufacture of lightweight, flexible, cost-effective electronic devices that are beyond the conventional silicon-based devices, including flexible displays [1], flexible and conformal antenna arrays [2], electronic solar cell arrays [3], radio-frequency identification tags [4], flexible batteries [5], electronic circuits fabricated in clothing [6], and biomedical devices [7]. Usually, most of them require electrical contacts.

Up to now, various materials such as conjugated polymers, graphene, carbon nanotubes, and metals have been used for the preparation of electrodes and conductive patterns using solution processing methods [8-11]. Specifically, metal nanoparticle inks have attracted more and more attention due to their high conductivity and thermal stability after having been sintered [12-14].

However, metallic nanoparticle inks often require high annealing temperatures (>150°C) to decompose stabilizing agents and other polymeric additives that inhibit electrical conductivity, with the high annealing temperature limiting the choice of substrate. Besides, they still cannot completely avoid the condensation and agglomeration of nanoparticles, especially after long-term storage. The agglomerated particles may damage the equipment and influence the printing quality. During preparation, a high-speed centrifuge or vacuum dryer must be used to take nanometal particles out, so these inks cannot be produced on a large scale. All of these will cause a higher production cost [15-18].

There is no surprise to the fact that organic silver conductive ink (OSC ink) has received increasing attention as a potentially much lower cost alternative [19-21]. This kind of ink mainly consists of a silver carrier, weak reduction agent, solvent, and additives, and a continuous conductive silver track can be fabricated during the sintering process. This strategy can compensate for the lack of conductive metal nanoink and thus becomes the development direction of conductive ink for macroelectronics [22-25].

In our previous research, the relationship between different kinds of amines and ink properties was investigated systematically. The addition of different amines not only increased the solid content of the conductive ink but also decreased the sintering temperature by complexation [26-28].

Here, based on the previous results, the formula of the conductive ink will be further optimized using silver acetate as silver carrier, ethanolamine as additive, and different kinds of organic aldehyde as reduction agents, such as ethylene glycol, acetaldehyde, formic acid, dimethylformamide, and glucose. Furthermore, the formula mechanism, conductive properties, temperature, dynamic fatigue properties, and feasibility verification of the OSC ink through the preparation of an antenna pattern were also investigated systematically [29-31].

## Methods

### Materials

Silver acetate was obtained from Shanghai Lingfeng Chemical Reagent Co., Ltd. (Shanghai, China). Polydimethylsiloxane (PDMS) including base and curing agents was obtained from Dow Corning Co. (Midland, MI, USA; SYLGARD 184 silicone elastomer). Polyester film (0.1 ± 0.02 mm) came from Shanghai Weifen Industry Co., Ltd (Shanghai, China). Ethylene glycol, acetaldehyde, formic acid, dimethylformamide, glucose, ethyl alcohol, and other solvents were of analytical grade and used without further purification. Deionized water was used in all experimental processes.

### Synthesis of OSC ink

For the preparation of conductive ink (1 g), silver acetate (0.32 g; which means if all silver ions are reduced to elemental silver, the content of elemental silver is 20 wt.%) and ethanolamine (0.2 g) were added to ethanol (0.13 g) and different reduction agents (0.35 g; ethylene glycol, acetaldehyde, formic acid, dimethylformamide, or glucose, etc.) under vigorous stirring until a transparent solution was obtained.

### Preparation of antenna pattern

For the preparation of the PDMS pattern as template, polyethylene terephthalate (PET) was adhered to a sheet glass using both side tapes, and 3-g PDMS (base/curing agent is 15/1) was dropped on the center of the PET film. Then, after spin coating (800 rpm), baking at 80°C for 3 h, and laser etching, the desired PDMS pattern as template can be fabricated with the conductive track (a thickness of 200 μm and a width of 200 μm).

For the preparation of the antenna pattern, the synthesized OSC ink was dropped into the trench of the PDMS template track using a syringe, and the ink will flow to all of the track spontaneously until full; then, it will be sintered at 120°C for 30 s. Finally, the PDMS template can be peeled off easily by forceps, and the desired antenna pattern was achieved [32].

### Instrumentation

OSC ink was characterized by using a Ubbelohde viscometer (CN60M, Zxyd Technology Co., Ltd., Beijing, China); a surface tension instrument (A101, Kino Industry Co., Ltd, New York, USA); X-ray diffraction (XRD; max 2550 PC, Rigaku-D, Rigaku Corporation, Tokyo, Japan) using Cu Kα radiation; scanning electron microscopy (SEM; S-360, Cambridge Instruments Ltd., Cambridge, England) operated at 10 kV; thermogravimetric analysis (TGA; QS-500, TA Instruments Inc., New Castle, DE, USA) with a heating rate of 5°C·min^−1^ in a nitrogen atmosphere; a four-point probe equipped with a semiconductor characterization system (BD-90, Shanghai Power Tool Institute, Shanghai, China); a memory hicorder (8870–20, HIOKI, Nagano, Japan) with a heating device from room temperature to 120°C, a steady current mechanism (10 mA), and an amplifier (×100); and a dynamic fatigue tester (built in the lab) with steady current mechanism (10 mA). The antenna pattern was investigated using a Uscan explorer with 3D profilometer system (D46047, Nanofocus, Oberhausen, Germany).

## Results and discussion

### Formula mechanism

**Figure 1:**
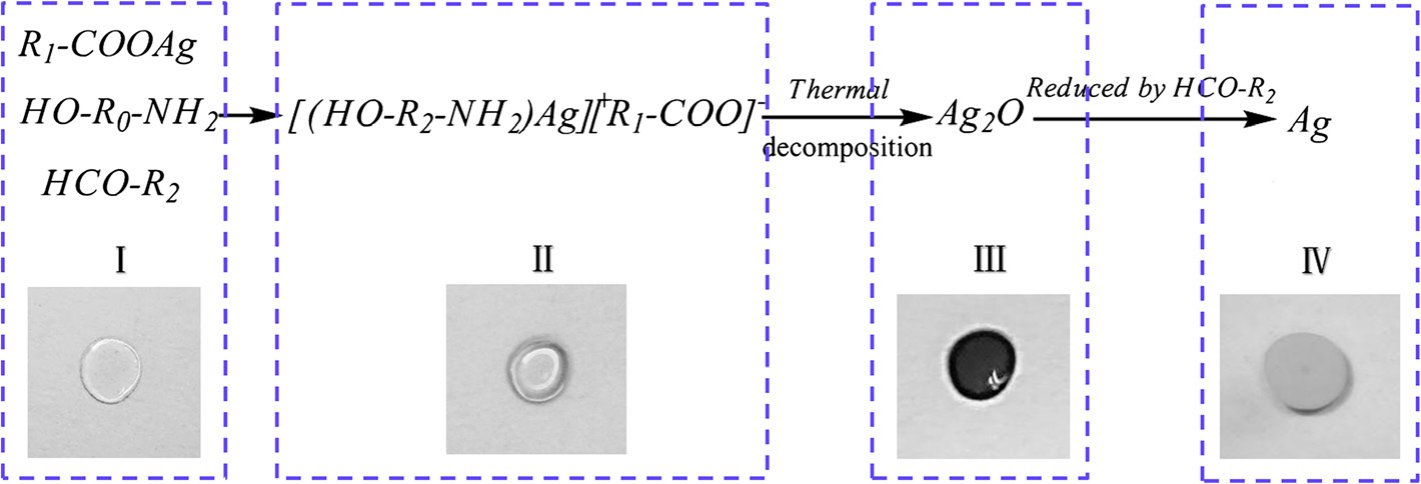
**Scheme of chemical reaction mechanism of OSC ink.** R_0_, R_1_, and R_2_ are carbon chains.

Compared with nanosilver conductive ink, the synthesized silver organic ink is transparent and clear without any visible particles. During the preparation process, this kind of conductive ink was mainly composed of a silver carrier, weak reduction agent, solvent, and additives. At the room temperature, it was very stable and can be kept for at least 1 month. Once it was heated, the complex chemical reaction occurred between the various components. Generally speaking, the sintering process can be divided into four stages: firstly, from simple silver ion to silver ion complex, then to silver oxide, and finally to elemental silver. Meanwhile, the color also changes from colorless to faint yellowish brown, to black, and to metallic luster. The details can be seen from Figure 1 directly.

In this formula, silver acetate was chosen as silver carrier, which can control the reaction rate effectively by adjusting the concentration of the silver ion in the mixing solvent because of its worse solubility. Ethanolamine was used to increase the silver content of the conductive ink to guarantee the conductivity and further to decrease the sintering temperature.

**Figure 2:**
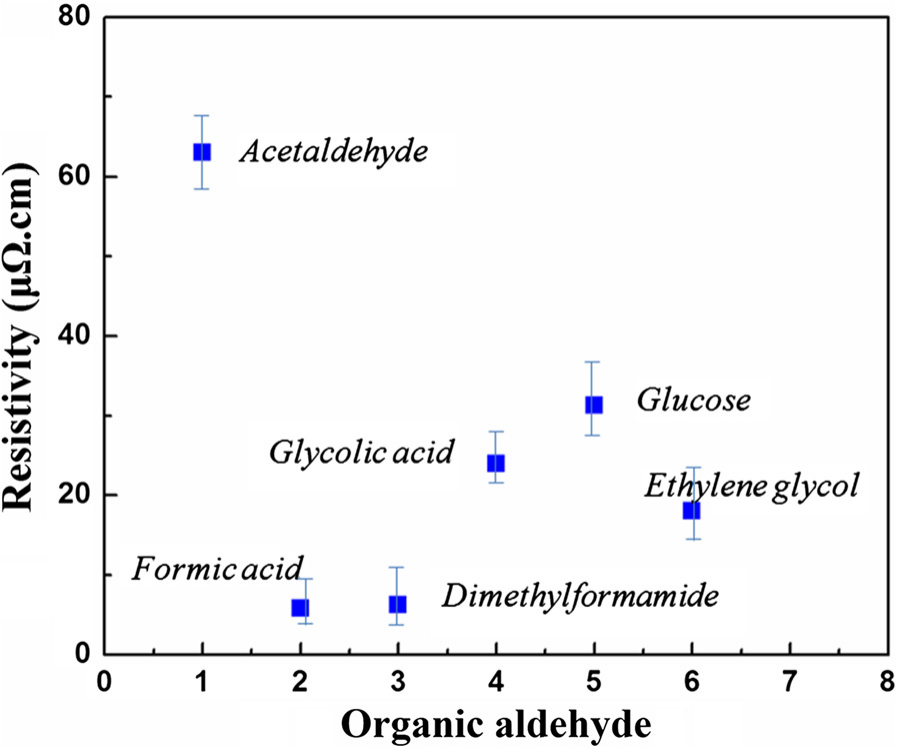
Resistivity of OSC ink (20 wt.%) with different reduction agents sintered at 120°C for 1 h.

Different aldehyde-based materials were chosen as weaker reduction agents, which have been discussed in detail as shown in Figure 2. Generally speaking, such materials can be divided into two types: one for itself with the aldehyde group, such as acetaldehyde, formic acid, dimethylformamide, and glucose; another for itself without the aldehyde group, but after heating, the aldehyde group can appear, such as ethylene glycol which can change to acetaldehyde at a high temperature and glycolic acid which can be decomposed into formaldehyde, carbon monoxide, and water at 100°C. The results show that reduction agent plays an important role on the properties of the conductive ink. Usually, a stronger reduction agent will bring in the instability of the ink, leading to the precipitation of silver particles and lower conductivity. Conversely, a weak reduction agent will result in a higher sintering temperature. It can be inferred that a suitable reduction agent is very important to get lower resistivity. From Figure 2, at the sintering temperature of 120°C for 1 h, the resisitivity of the silver thin film with different formulas should be very stable. It can be seen that formic acid and dimethylformamide show lower resistivity of about 6 to 8 μΩ·cm and 7 to 9 μΩ·cm, respectively. In view of the formula stability, in the following research, dimethylformamide was chosen as the reduction agent.

### OSC ink properties

**Figure 3:**
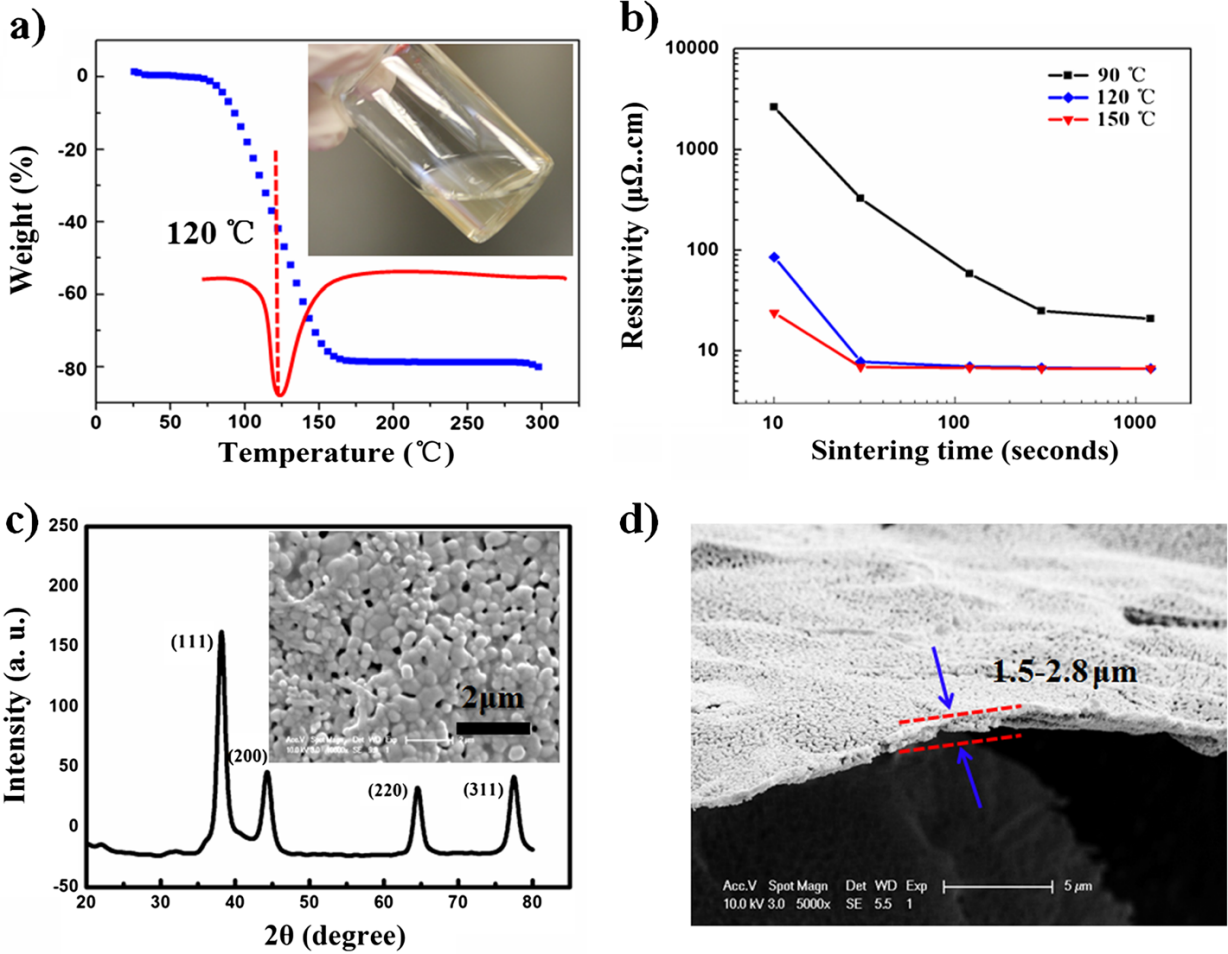
**Ink properties.** (**a**) TGA and DTG curves (inset, OSC ink). (**b**) Variation of resistivity sintered at different temperatures for different times. (**c**) XRD pattern of sintered OSC ink with a solid content of 20 wt.% (the inset shows the top-view SEM image of the conductive film). (**d**) Lateral view of the SEM image of the silver film sintered at 120°C for 30 s (dimethylformamide was used as reduction agent in the formula).

For further investigation of the OSC ink, dimethylformamide was used as reduction agent in the formula. The viscosity and surface tension were adjusted to 13.8 mPa·s and 36.9 mN/m (20°C), which can totally fulfill the requirement of ink-jet printing, as shown in the inset of Figure 3a.

The thermal properties of the prepared OSC ink were investigated by TGA with a heating rate of 5°C/min, as depicted in Figure 3a. It can be seen that there exists an evident mass-decreasing area, from 80°C to 160°C, which is related to the evaporation of organic materials; finally, 20.3 wt.% of the mass remains, which indicates that the ink contains 20.3 wt.% silver and agrees well with the calculated value (20 wt.%). If several drops of ammonia were added, the solid content can be further increased to 28 wt.% at most because of its stronger coordination ability than ethanolamine. However, more ammonia will cause the instability of the conductive ink due to its volatilization.

The conductive properties of the prepared OSC ink were investigated using different sintering temperatures (90°C, 120°C, 150°C) for different durations of time (from 0 to 60 min), which also can be explained by percolation theory, as shown in Figure 3b. During the sintering process, initially, there are only silver acetate and silver oxide, without any elemental silver, so there is no conductivity. Then, almost all of the silver oxide was reduced to elemental silver at the same time, indicating that a continuous conductive track has been fabricated and showing metallic luster and high conductivity. Especially, based on the present formula of the ink, when the sintering temperature is 120°C for 30 s, the resistivity can drop to 7 to 9 μΩ·cm.

Figure 3c shows an XRD pattern of the silver ink after sintering, and all diffraction peaks could be indexed to the face-centered cubic phase of silver. The lattice constant calculated from this XRD pattern was 4.098, which was very close to the reported data (*a* = 4.0862, JCPDS file no. 04–0783). The inset is the surface morphology of the conductive ink after sintering, and more information also can be seen from Figure 3d.

### Temperature and dynamic fatigue properties

**Figure 4:**
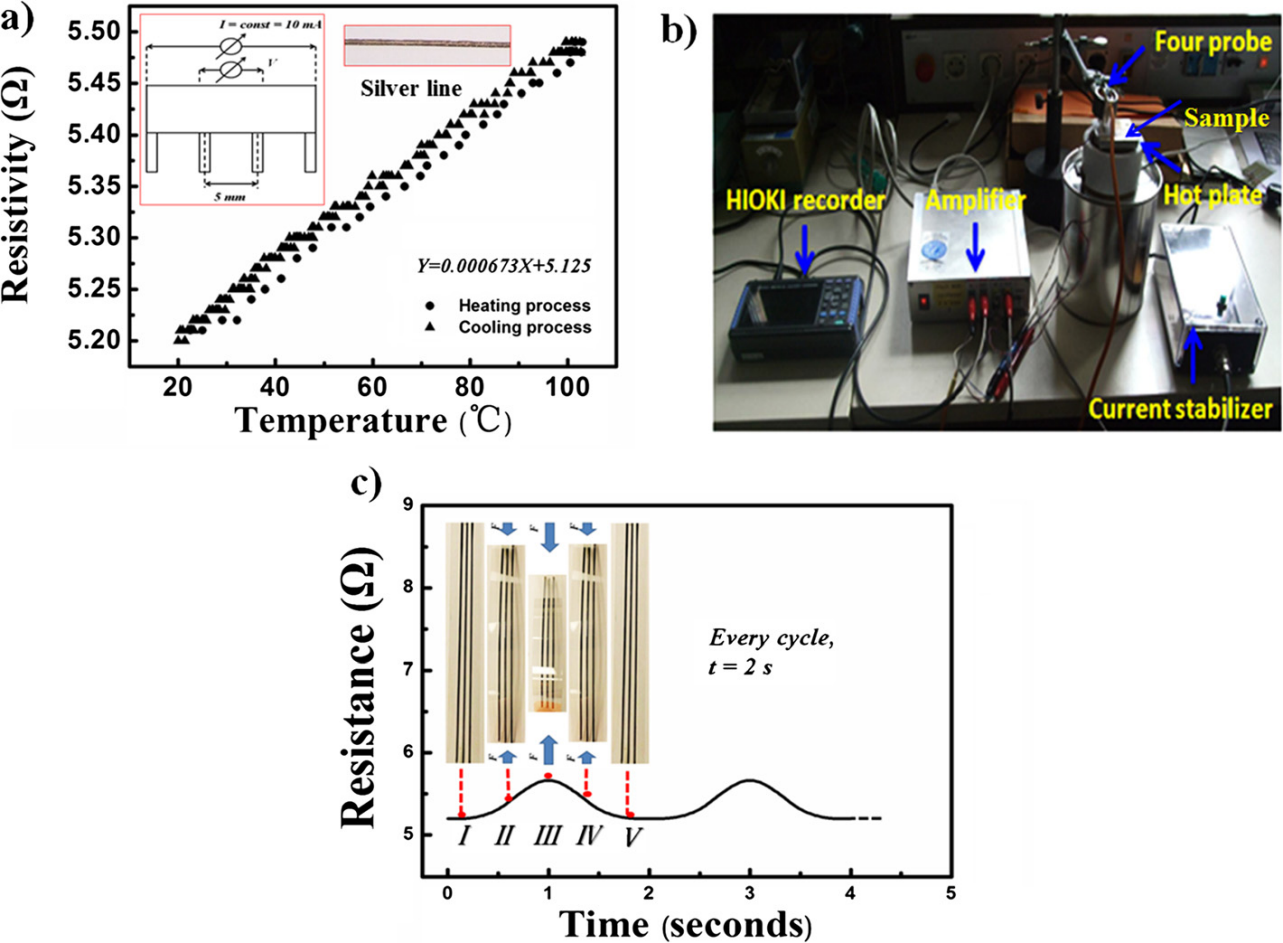
**Correlations between resistivity and temperature, and dynamic fatigue of the conductive silver line.** (**a**) Relationship and (**b**) measurement equipment of resistance versus the change of the temperature. (**c**) Dynamic fatigue properties of PET-based conductive patterns sintered at 120°C for 30 s.

To verify the applicability of this approach in macroelectronics, the correlations between resistivity and temperature, and dynamic fatigue of the conductive silver line were investigated systematically, which were shown in Figure 4.

From Figure 4a,b, a set of equipment including a heating device from room temperature to 120°C, steady current mechanism (10 mA), amplifier (×100), memory hicorder (HIOKI, 8870–20), etc. were assembled together, aiming at monitoring the changes of the resistivity of the conductive silver line during the heating and cooling processes. It can be obtained that between 20°C and 100°C, the largest variable quantity of the resistivity is just about 0.28 Ω. After linear fitting, the slopes of the heating curve and the cooling curve, which can be called temperature coefficient of resistance (TCR), approximately have the same slope (kh = kc = 0.0007 aR/°C^−1^), indicating the good thermal stability of the conductive silver line. The TCR is a little different compared with the TCR of bulk silver (0.0038 aR/°C^−1^). This phenomenon is mainly caused by the complex microstructure of the silver thin film which will bring more barriers during the electron-transfer process. Moreover, it also can be seen that though the heating curve and cooling curve have the same TCR, the cooling curve is always below the heating curve. This is mainly because the natural cooling process (about 28 min) needs more time than the heating process (15 min).

From Figure 4c, a bending tester was used to study the dynamic fatigue of the PET-based conductive silver line. During the test, the conductive line makes a periodic bending movement from I to V, and every period needs 2 s. The details also can be seen from the set in Figure 3b. It is very interesting to find that the resistivity of the conductive silver lines also increases with the increase of the bending angle. From I to III, the resistivity increases from 5.2 to 5.76 Ω. It can be explained that when bending, the silver thin film was stretched and became thin, especially on the top point of the conductive line, so the stack density and conductivity decreased. From III to V, the resistivity was back to 5.2 Ω, and after a periodic movement like this for 1,000 times, the resistivity did not significantly increase due to the good ductility of the metal silver. Generally speaking, compared with other printing technologies, this method also shows good adhesion between the silver thin film and PET, showing good results.

### Preparation of an antenna pattern

**Figure 5:**
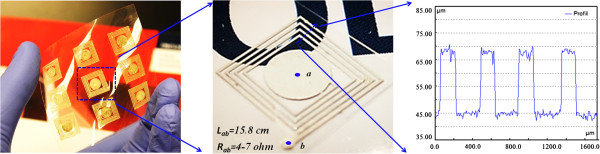
**Antenna pattern after sintering at 120°C for 30 s and surface profile curves of conductive pattern.** The prepared antenna pattern was fabricated using drop or fit-to-flow method.

To test the practical applications of the prepared OSC ink here, an antenna pattern (11 mm × 12 mm) was designed and fabricated using fit-to-flow or drop method, which also can be seen from Figure 5 directly.

The thin film PDMS pattern with a thickness of 200 μm, a width of 200 μm, and a total length of 15.8 cm on the PET substrate was prepared using a laser and used as template. The synthesized OSC ink with blue dye (seen more clearly) was dropped to the center of the template using a syringe (20 μL per drop). Due to the good wetting and film-forming ability of the ink, it will flow along the template track until it fills the whole track, especially after plasma treatment with oxygen. After sintering at 120°C for 30 s, the continuous conductive track can be fabricated, and the total resistor *R*_ab_ decreased to 4.6 Ω measured by a multimeter (middle image of Figure 5) with a width of 200 μm and thickness of 22 μm according to the surface profile.

## Conclusions

In summary, an unusual kind of high-efficiency, transparent organic silver conductive ink (OSC ink) was synthesized with silver acetate as silver carrier, ethanolamine as additive, and different kinds of aldehyde-based materials as reduction agents successfully. The results show that different reduction agents have an important influence on the ink properties through a series of complex chemical reactions, and when formic acid or dimethylformamide was used as the reduction agent and sintered at 120°C for 30 s, the resistivity can be lowered down to 6 to 9 μΩ·cm. It also can be obtained that the fabricated conductive pattern shows good temperature and dynamic fatigue properties. Besides, the feasibility of the synthesized OSC ink was verified through the preparation of an antenna pattern using drop or fit-to-flow method successfully.

## Competing interests

The authors declare that they have no competing interests.

## Authors’ contributions

Y-LT synthesized the organic silver conductive ink and discussed the formula. YT, LW, YT, and BW characterized and investigated the properties of the OSC ink. All authors took part in the writing of the manuscript and approved the final manuscript.
